# Oncoplastic versus conventional breast-conserving surgery in breast cancer: a pooled analysis of 6941 female patients

**DOI:** 10.1007/s12282-022-01430-5

**Published:** 2023-01-09

**Authors:** Mohammed Tarek Hasan, Mohamed Hamouda, Mohammad. K. El khashab, Ahmed Bostamy Elsnhory, Abdullah Mohamed Elghamry, Obada Atef Hassan, Aya Mamdouh Fayoud, Abdelrahman H. Hafez, Mohammed Al-kafarna, Abdulrahman Ibrahim Hagrass, Randa Kamal Rabea, Mohamed Ibrahim Gbreel

**Affiliations:** 1 Faculty of Medicine for Boys, Al-Azhar University, Cairo, Egypt; 2 Faculty of Pharmacy, Kafr El-Shaikh University, Kafr El-Shaikh, Egypt; 3 Faculty of Pharmacy, Al-Azhar University–Gaza, Gaza Strip, Palestine; 4 Faculty of Medicine, Ain Shams University, Cairo, Egypt; 5 Faculty of Medicine, October 6 University, Cairo Governorate, Giza, 11571 Egypt; 6 International Medical Research Association (IMedRA), Cairo, Egypt

**Keywords:** Breast cancer, Oncoplastic surgery, Breast-conserving surgery, Meta-analysis

## Abstract

**Background:**

Breast cancer is the most prevalent cancer in women. In the past few years, surgical interventions for breast cancer have experienced massive changes from radical excision to conserving approaches. In this study, we aim to compare the two breast surgery interventions, including conventional breast-conserving surgery (CBCS) versus oncoplastic breast-conserving surgery (OPBCS).

**Methods:**

We searched on PubMed, Web of Science (WOS), Scopus, Embase, and Cochrane till 2 October 2021. All relevant randomized controlled trials (RCTs) and observational studies were included. The data were extracted and pooled using Review Manager software (RevMan 5.4).

**Results:**

The pooled meta-analysis of the included studies showed that OPBCS was significantly superior to CBCS in most of the outcomes. Re-excision significantly favoured CBCS (RR = 0.49, 95% CI [0.37, 0.63], *P* < 0.00001). However, local recurrence (RR = 0.55, 95% CI [0.27, 1.09], *P* = 0.09), close surgical margins (RR = 0.37, 95% CI [0.14, 1.00], *P* = 0.05) and end up to the risk of mastectomy (RR = 0.73, 95% CI [0.54, 97], *P* = 0.06) showed no significant difference between both techniques. Notably, while performing a sensitivity analysis, other outcomes as local recurrence, significantly showed favourable results towards OPBCS. In terms of safety outcomes, there was no significant difference between OPBCS and CBCS.

**Conclusion:**

We recommend the oncoplastic approach rather than the conventional one in females with breast cancer. Re-excision rates showed better results following OPBCS.

**Supplementary Information:**

The online version contains supplementary material available at 10.1007/s12282-022-01430-5.

## Introduction

One of the most prevalent cancers in women worldwide is breast cancer, accounting for 25% of all cancers amongst women and 14% of all deaths related to cancer [1, 2]. Over the past years, surgical intervention has experienced a continuous and massive change, shifting from radical procedures toward more patient-satisfying breast-conserving approaches [3, 4].

Conventional Breast-conserving surgery (CBCS) coupled with postoperative radiation has been the primary locoregional management for most early-stage cases, with a survival rate equal to that of a mastectomy, surgery of removing one or both breasts [5]. CBCS’s success depends on complete cancer removal with sufficient surgical margins to ensure that the specimen is clear of the tumour while keeping the breast’s natural look and shape, improving patient satisfaction and body image. Achieving both targets in the same procedure is difficult, and CBCS does not yield satisfactory cosmetic outcomes in all cases [6, 7]. One of the main criteria that limits the quantity of tissue that may be removed is not only the absolute breast volume but also the proportion to the tumour’s location and the breast’s dimensions. If neither of these aims can be met, the patient is frequently directed to mastectomy. Another option is to use chemotherapy or hormone treatment to shrink the tumour pre-operative. However, neoadjuvant therapy does not work for all tumours. The inability of conventional CBCS to solve these challenges has encouraged the development of new breast surgery techniques, such as oncoplastic breast surgery [8].

Oncoplastic breast surgery (OBS) is a new trend in CBCS that merges oncology and plastic operation concepts to achieve both oncological and aesthetic satisfying outcomes [9]. Moreover, OBS enables the removal of significantly larger tumours as it has become a non-mastectomy option in tumours larger than 4 cm and locoregional tumours [10]. However, OBS minimizes the necessity of subsequent correction deformities, which can lead to delayed healing and poor cosmetic result, particularly when radiation is used after surgery [11]. The promising outcomes reported about OBS encourage some experts to consider OBS the standard care.

Hence, the meta-analysis is a statistical method for collecting the findings of numerous studies on a single topic and resolving discrepancies; we aim to compare both two breast surgery conserving interventions, including conventional breast-conserving surgery (CBCS) versus oncoplastic breast-conserving surgery (OPBCS).

## Materials and methods

We followed the approaches for conducting the current study based on the Cochrane handbook of systematic reviews on interventions [12]. During the drafting of our manuscript, we strictly followed the recommended reporting items for the Preferred Reporting Items for Systematic Reviews and Meta-Analyses (PRISMA) statement guidelines [13].

### Search strategy

The following electronic databases were systematically searched: PubMed, Web of Science, Scopus, Embase, and Cochrane till 2 October 2021. We used the following Mesh terms to find our results: oncoplastic and Conventional and Breast. The screening was also performed on the references of the included studies and pertinent reviews to avoid missing any studies and guarantee high-quality screening.

### Eligibility criteria

We included all articles that matched the following requirements: (1) population: patients undergoing breast surgery, (2) intervention: oncoplastic or conservative surgery, (3) comparison: conventional surgery, (4) study design: randomized clinical trials (RCTs), cohort and case–control studies. We excluded non-human studies, conference abstracts, and non-English studies.

### Studies selection

We used Endnote software to remove duplicates, and the retrieved references were screened to assess their relevance. The screening was done in two steps; title and abstract screening, followed by full-text screening for final eligibility. Each step was done at least by two independent authors, and the findings were compared, and group discussions then solved disagreements.

### Quality assessment

For all RCTs that were included, the Cochrane collaboration tool was used to evaluate their quality [12]. It encompasses the following domains: randomization, concealment of allocation, blinding of participants and workers, blinding of outcome assessment, incomplete outcome data, selective reporting, and other sources of bias. The evaluation is based on a determination of whether there is a low, high, or unclear bias risk. For the quality assessment of observational studies, we used the Newcastle–Ottawa Scale (NOS) [14]. It includes selection, comparability, and exposure. Each domain is assessed using stars, with a maximum of nine stars.

### Data extraction

Two independent authors extracted the following data from the included studies: (1) summary of included studies: title, study design, country, participants and key inclusion/exclusion criteria, intervention group, control group, and conclusion; (2) baseline characteristics of the enrolled participants: age, gender, BMI, menopausal status, tumour size and grade, and histopathology. Disagreements were solved later by group discussion.

### Primary and secondary endpoints

In this study, we concentrated on the following postoperative parameters: re-excision, local recurrence, dissected lymph nodes, positive surgical margin, negative surgical margin, close surgical margins, mastectomy, distant metastasis, reoperation, radiotherapy, chemotherapy, endocrine therapy, immune therapy, ipsilateral breast tumour recurrence, surgical time (min) and volume of the specimen (in cm^3^).

### Statistical analysis

We used the Review Manager software (RevMan 5.4). Dichotomous data were analyzed as odds ratio (OR) and 95% confidence interval (CI) and continuous data as mean difference (MD) and 95% CI. Statistical heterogeneity among the studies was assessed by visual inspection of the forest plot, besides using I-squared (*I*^2.^) and chi-squared (Chi^2^) statistics. *I*^2^ values of 50% were indicative of high heterogeneity [15, 16]. A random-effects model was applied when there was a significant variation in the data. Other than that, the fixed-effect model was applied.

## Result

In our SR and MA, we analyzed 14 studies with 6941 patients, 2253 of them were in the OPBCS group, and 4688 were in the CBCS group.

### Literature search

**Fig. 1:**
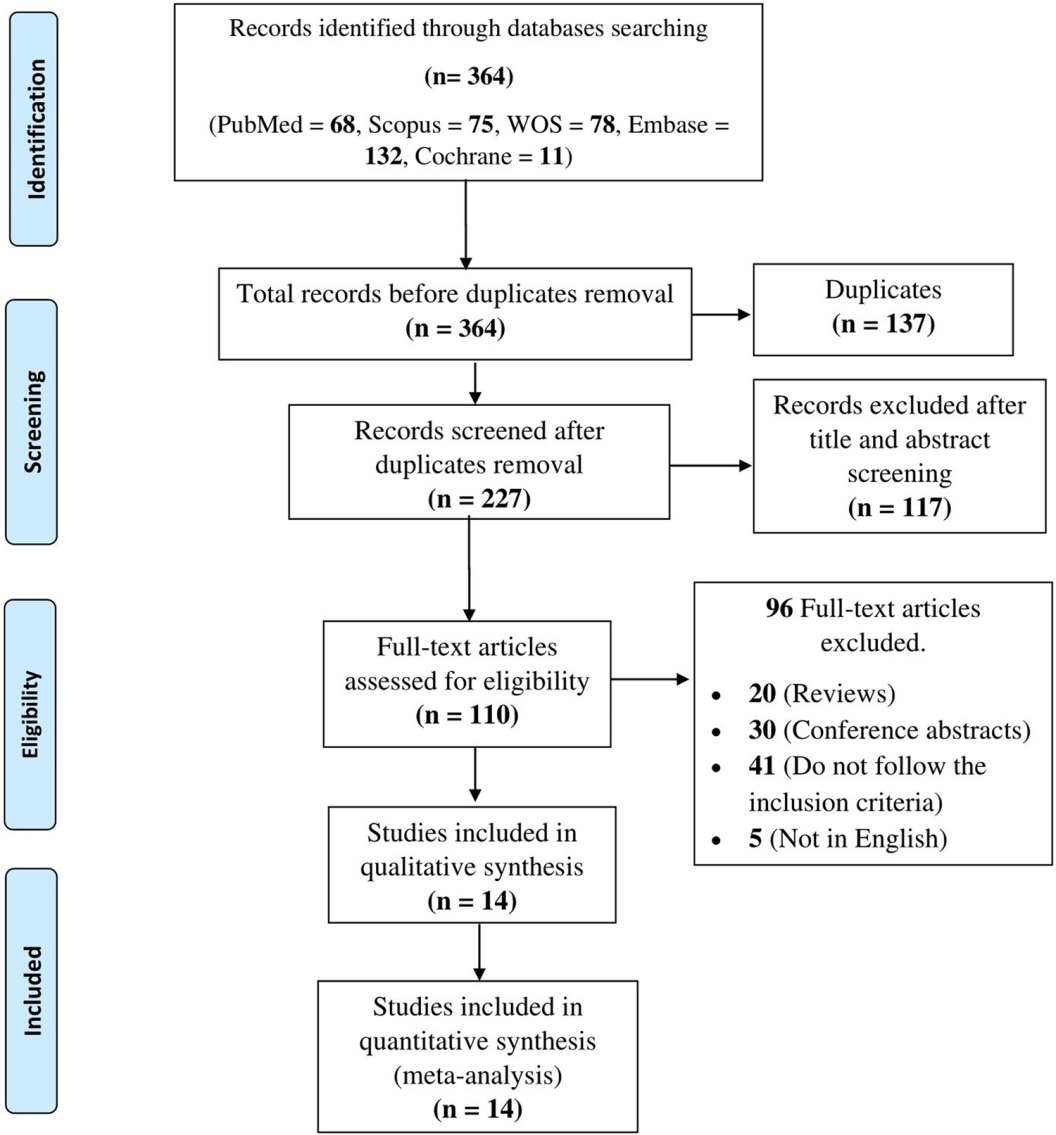
PRISMA flow diagram showing the literature search results

The initial search results in 364 articles from the five databases: 68 from PubMed, 11 from Cochrane CENTRAL, 75 from Scopus, 78 from Web of Science, and 132 from Embase. Of these 364 articles, we excluded 137 articles due to duplications, and 227 articles underwent title and abstract screening. We excluded 117 as they did not meet our inclusion criteria. The remaining 110 papers were subjected to a full-text review. Finally, 14 studies were included (Fig. 1. PRISMA flow diagram).

### Demographics and characteristics

The present study included 6941 patients from 14 trials that matched our criteria for inclusion [14, 17–29]. Except for Dogru et al. 2018 [22], where the control group was conventional excisional biopsy, all studies included a comparison of OPBCS (2253 patients) versus CBCS (4688 patients). Twelve of the studies were cohort studies, one was a case–control study, and one was an RCT. The studies we looked at were conducted in ten different countries. Tables 1, 2 provide the baseline and summary of the included studies.

**Table 1:** Summary of the included studies

Study ID	Year	Setting	Design	Sample size	
				OBCS	CBCS
Atallah et al.	2021	Lebanon	Case-control study	193	84
Behluli et al.	2019	Germany	Cohort	291	52
Bromberg et al.	2018	Brazil	Cohort	34	26
Chauhan et al.	2016	India	Randomized controlled study	33	46
Doğru et al.	2018	Turkey	RCT	40	40
Dogan et al.	2021	Turkey	Cohort	47	142
Gulcelik et al.	2013	Turkey	Cohort	106	162
Kelemen et al.	2018	Hungary	Cohort	350	350
Mátrai et al.	2014	Hungary	Cohort	59	60
Niinikoski et al.	2019	Finland	Cohort	611	1189
Oberhauser et al.	2020	Switzerland	Cohort	188	95
Rose et al.	2019	Denmark, Sweden	Cohort	197	1399
Rose et al.	2020	Denmark, Sweden	Cohort	96	631
Sakina et al.	2021	Pakistan	Cohort	249	173

**Table 2:** Baseline characteristics of the included studies

Study ID	Sample	Study arms	Age (years), (Mean ± SD)	BMI (Kg/m^2^), (Mean ± SD)	Menopausal status, *N* (%)	Tumor size (cm), (Mean ± SD)	Tumor volume (cm^3^), (Mean ± SD)	Grade, *N* (%)		
								1	2	3
Atallah 2012	193	OBCS	53.56 ± 12.52	26.32 ± 5.31	93 (48.2%)	1.89 ± 1.11	5.94 ± 3.50	26 (13.5)	79 (40.9)	34 (17.6)
	84	CBCS	52.07 ± 11.47	25.46 ± 6.13	30 (35.7%)	1.86 ± 0.95	5.85 ± 3.01	13 (15.5)	23 (27.4)	18 (21.4)
Behluli 2019	52	OBCS	62 ± 86.53		–	2.4 ± 8.653	–	5 (9.6)	26 (50)	21 (40.4)
	291	CBCS	59 ± 187.65	–	–	2.5 ± 30.706	–	66 (22.7)	142 (48.8)	83 (28.5)
Bromberg 2018	34	OBCS	53.9 ± 10.75	–	–	–	–	30 (88)	4 (12)	0
	26	CBCS	57.4 ± 12.75	–	–	–	–	21 (81)	5 (19)	0
Chauhan2016	57	OBCS	46.9 ± 13.1	–	–	–	5.3 ± 1.2	13 (23%)	31 (54%)	13 (23%)
	43	CBCS	54.3 ± 5.3	–	–	–	4.9 ± 1.3	9 (27%)	18 (42%)	14 (31%)
Doğru 2018	40	OBCS	43.3 ± 11.2	–	15 (37.5)	–	9.61 ± 6.54	–	–	–
	40	CBCS	48.4 ± 13.7	–	22 (55)	–	9.98 ± 8.34	–	–	–
Dogan 2021	47	OBCS	48 ± 8	31 ± 4	–	3.6 ± 12	–	–	–	30 (64%)
	142	CBCS	49 ± 7	27 ± 3	–	2.4 ± 8	–	–	–	85 (60%)
Gulcelik 2013	106	OBCS	53 ± 12.4	–	–	2.7	–	–	–	–
	162	CBCS	51 ± 10.8	–	–	2.4	–	–	–	–
Kelemen2018	350	OBCS	58 ± 9	23.3 ± 2.22	–	–	–	107 (30.6)	127 (36.3)	89 (25.4)
	350	CBCS	59 ± 9.5	22.9 ± 2.4	–	–	–	94 (26.9)	139 (39.7)	87 (24.9)
Mátrai 2014	60	OBCS	57.2 ± 10.27	–	–	–	–	9 (15)	31 (51.7)	20 (33.3)
	60	CBCS	57.1 ± 10.76	–	–	–	–	18 (30.5)	20 (33.9)	21 (35.6)
Niinikoski 2019	611	OBCS	61 ± 8.17	–	–	1.2 ± 1.42	–	175 (28.6)	237 (38.8)	198 (32.4)
	1189	CBCS	62 ± 9.67	–	–	12 ± 14.17	–	416 (35)	500 (42.1)	272 (22.9)
Oberhauser 2020	188	OBCS	60.33 ± 14.94	–	–	–	–	46 (24.5)	81 (43.1)	57 (30.3)
	95	CBCS	61.67 ± 15.81	–	–	–	–	24 (25.3)	44 (46.3)	21 (22.1)
Rose 2019	197	OBCS	–	–	64 (32.50)	–	–	49 (24.90)	83 (42.10)	53 (26.90)
	1399	CBCS	–	–	283 (20.20)	–	–	414 (29.60)	506 (36.20)	297 (21.20)
Rose 2020	69	OBCS	–	–	61 (63.5)	–	–	–	–	–
	631	CBCS	–	–	466 (73.90)	–	–	–	–	–
Sakina 2021	249	OBCS	49.9	–	–	2.26 ± 1.66	–	–	–	–
	173	CBCS	51.1	–	–	1.94 ± 1.28	–	–	–	–

Study ID	Histopathology, *N* (%)						Location in breast, *n* (%)						
	IDC	ILC	IDLC	DCIS	Mixed*	Other	A	B	C	D	E	F	G
Atallah 2012	105 (66)	53 (33.3)	1 (0.6)	24 (13)	–	7 (3.8)	64 (33.5)	26 (13.6)	23 (12)	9 (4.7)	22 (11.5)	9 (4.7)	38 (19.9)
	37 (60.7)	23 (37.7)	1 (0.6)	23 (37.7)	–	7 (8.4)	37 (47.4)	8 (10.3)	7 (9)	3 (3.8)	14 (17.9)	2 (2.6)	7 (9)
Behluli 2019	–	28 (54)	–	8 (15)	16 (31)	–	–	–	–	–	–	–	–
	–	165 (57)	–	48 (16)	78 (27)	–	–	–	–	–	–	–	–
Bromberg 2018	–	–	–	–	–	–	16 (47)	4 (12)	9 (26)	4 (12)	0	0	1 (3)
	–	–	–	–	–	–	11 (42)	7 (27)	4 (15)	1 (4)	0	0	3 (12)
Chauhan2016	54 (94%)	1 (2%)	–	–	–	2 (4%)	33 (57.9)	7 (12.3)	5 (8.8)	4 (7)	–	–	8 (14)
	42 (96%)	1 (2%)	–	–	–	1 (2%)	34 (79.1)	5 (11.5)	2 (4.7)	2 (4.7)	–	–	0
Doğru 2018	–	5 (12.5)	–	3 (7.5)	–	–	–	–	–	–	–	–	–
	–	5 (12.5)	–	2 (5)	–	–	–	–	–	–	–	–	–
Dogan 2021	–	–	–	–	–	–	–	–	–	–	–	–	–
	–	–	–	–	–	–	–	–	–	–	–	–	–
Gulcelik 2013	–	–	–	–	–	–	–	–	–	–	–	–	–
	–	–	–	–	–	–	–	–	–	–	–	–	–
Kelemen2018	271 (77.4)	35 (10)	–	170 (48.6)	–	22 (6.3)	–	–	–	–	–	–	–
	277 (79.1)	21 (6)	–	124 (35.4)	–	22 (6.3)	–	–	–	–	–	–	–
Mátrai 2014	–	–	–	–	–	–	26 (43.4)	12 (20)	11 (18.3)	4 (6.7)	3 (5%)	1 (1.6)	3 (5)
	–	–	–	–	–	–	36 (60)	7 (11.7)	11 (18.3)	6 (10)	0	0	0
Niinikoski 2019	424 (69.4)	76 (12.4)	–	29 (4.7)	–	82 (13.4)	–	–	–	–	–	–	–
	826 (69.5)	136 (11.4)	–	64 (5.4)	–	163 (13.7)	–	–	–	–	–	–	–
Oberhauser 2020	–	–	–	23 (12.2)	–	–	–	–	–	–	–	–	–
	–	–	–	11 (11.6)	–	–	–	–	–	–	–	–	–
Rose 2019	171 (86.8)	14 (7.1)	–	–	–	11 (5.6)	–	–	–	–	–	–	–
	1142 (81.6)	101 (7.2)	–	–	–	146 (10.4)	–	–	–	–	–	–	–
Rose 2020	–	–	–	–	–	–	39 (40.60)	8 (8.30)	9 (9.40)	6 (6.3)	–	–	15 (15.6)
	–	–	–	–	–	–	233 (36.90)	92 (14.60)	56 (8.90)	44 (7)	–	–	39 (6.2)
Sakina 2021	193 (84.3%)	2 (0.9%)	–	–	–	–	121 (48.8%)	27 (10.9%)	46 (18.5%)	23 (9.3%)	–	–	31 (12.5%)
	105 (76.1%)	2 (1.4%)	–	–	–	–	90 (50.2%)	27 (15.6%)	28 (16.2%)	17 (9.8%)	–	–	11 (6.4%)

Study ID	Number	Study arm	TNM staging, *N* (%)										
			Tis	T0	T1	T2	T3	T4	ypT0	ypT1a	ypT1b	ypT1c	ypT2
Atallah 2012	193	OBCS	–	–	–	–	–	–	–	–	–	–	–
	84	CBCS	–	–	–	–	–	–	–	–	–	–	–
Behluli 2019	52	OBCS	8 (15.4)	–	21 (40.4)	22 (42.3)	1 (1.9)	0	–	–	–	–	–
	291	CBCS	48 (16.5)	–	156 (53.6)	73 (25.1)	14 (4.8)	0	–	–	–	–	–
Bromberg 2018	34	OBCS	–	–	–	–	–	–	–	–	–	–	–
	26	CBCS	–	–	–	–	–	–	–	–	–	–	–
Chauhan2016	57	OBCS	–	–	14 (24%)	26 (47%)	9 (16%)	8 (13%)	–	–	–	–	–
	43	CBCS	–	–	11 (25%)	27 (63%)	3 (7%)	2 (5%)	–	–	–	–	–
Doğru 2018	40	OBCS	–	–	–	–	–	–	–	–	–	–	–
	40	CBCS	–	–	–	–	–	–	–	–	–	–	–
Dogan 2021	47	OBCS	–	–	–	–	–	–	–	–	–	–	–
	142	CBCS	–	–	–	–	–	–	–	–	–	–	–
Gulcelik 2013	106	OBCS	–	–	–	–	–	–	–	–	–	–	–
	162	CBCS	–	–	–	–	–	–	–	–	–	–	–
Kelemen2018	350	OBCS	22 (6.3)	–	210 (60)	88 (25.1)	2 (0.6)	1 (0.3)	15 (4.3)	9 (2.6)	–	2 (0.6)	1 (0.3)
	350	CBCS	30 (8.6)	–	226 (64.6)	91 (26)	1 (0.3)	1 (0.3)	0	1 (0.3)	–	0	0
Mátrai 2014	60	OBCS	4 (6.7%)	–	–	22 (36.7%)	–	–	–	1 (1.6%)	6 (10%)	27 (45%)	–
	60	CBCS	5 (8.3%)	–	–	6 (10%)	–	–	–	5 (8.3%)	19 (31.7%)	25 (41.7%)	–
Niinikoski 2019	611	OBCS	28 (4.6)	–	415 (67.9)	164 (26.8)	3 (0.5)	1 (0.2)	–	–	–	–	–
	1189	CBCS	64 (5.4)	–	990 (83.3)	132 (11.1)	2 (0.2)	1 (0.1)	–	–	–	–	–
Oberhauser 2020	188	OBCS	24 (12.8)	8 (4.3)	109 (58.0)	40 (21.3)	6 (3.2)	0	–	–	–	–	–
	95	CBCS	11 (11.6)	6 (6.3)	60 (63.2)	18 (18.9)	0	0	–	–	–	–	–
Rose 2019	197	OBCS	–	–	–	–	–	–	–	–	–	–	–
	1399	CBCS	–	–	–	–	–	–	–	–	–	–	–
Rose 2020	69	OBCS	–	–	–	–	–	–	–	–	–	–	–
	631	CBCS	–	–	–	–	–	–	–	–	–	–	–
Sakina 2021	249	OBCS	–	–	–	–	–	–	–	–	–	–	–
	173	CBCS	–	–	–	–	–	–	–	–	–	–	–

Study ID	Lymph nodes, *N* (%)									Hormone receptors, *N* (%)										
	NX	N0	N1	N2		N3	ypN0	ypN1	ypN2a	ER +	ER−	PR +	PR−	HER2 +	HER2−	Triple negative	Ki67 ≥ 20%	Luminal A-like	Luminal B-like (Her2-negative)	Luminal B-like (Her2-positive)
Atallah 2012	–	–	–		–	–	–	–	–	140 (89.7)	–	136 (88.3)	–	10 (6.7)	–	–	–	–	–	–
	–	–	–		–	–	–	–	–	52 (85.2)	–	56 (91.8)	–	4 (6.8)	–	–	–	–	–	–
Behluli 2019	–	38 (80.85)	4 (8.51)		4 (8.51)	0	–	–	–	42 (84)	–	30 (60)	–	–	–	–	–	–	–	–
	–	192 (79)	41 (16.9)		6 (2.5)	4 (1.6)	–	–	–	248 (86)	–	207 (72)	–	–	–	–	–	–	–	–
Bromberg 2018	–	–	–		–	–	–	–	–	–	–	–	–	–	–	–	–	–	–	–
	–	–	–		–	–	–	–	–	–	–	–	–	–	–	–	–	–	–	–
Chauhan2016	–	21 (37%)	29 (51%)		7 (12%)	–	–	–	–	32 (56%)	25 (44%)	–	–	21 (37%)	36 (63%)	–	–	–	–	–
	–	14 (32%)	27 (61%)		2 (7%)	–	–	–	–	27 (63%)	16 (37%)	–	–	19 (45%)	24 (55%)	–	–	–	–	–
Doğru 2018	–	–	–		–	–	–	–	–	–	–	–	–	–	–	–	–	–	–	–
	–	–	–		–	–	–	–	–	–	–	–	–	–	–	–	–	–	–	–
Dogan 2021	–	–	–		–	–	–	–	–	35 (75%)	–	31 (66%)	–	–	–	–	–	–	–	–
	–	–	–		–	–	–	–	–	112 (79%)	–	88 (62%)	–	–	–	–	–	–	–	–
Gulcelik 2013	–	–	–		–	–	–	–	–	77 (72.6)	–	–	–	24 (22.6)	–	–	–	–	–	–
	–	–	–		–	–	–	–	–	80 (49.3)	–	–	–	21 (12.9)	–	–	–	–	–	–
Kelemen2018	–	237 (67.7)	67 (19.2)		16 (4.6)	3 (0.9)	17 (4.9)	7 (2)	3 (0.9)	271 (77.4)	–	119(34)	–	19 (5.4)	–	38 (10.9)	130 (37.1)	–	–	–
	–	317 (90.6)	25 (7.1)		6 (1.7)	1 (0.3)	0	1 (0.3)	0	303 (86.6)	–	143 (40.9)	–	5 (1.4)	–	12 (3.4)	79 (22.6)	–	–	–
Mátrai 2014	–	51 (85%)	9 (15%)		–	–	–	–	–	46 (76.7%)	–	42 (70%)	–	6 (10%)	–	–	–	–	–	–
	–	48 (80%)	12 (20%)		–	–	–	–	–	51 (85%)	–	43 (71.7%)	–	3 (5%)	–	–	–	–	–	–
Niinikoski 2019	–	415 (67.9)	196 (32.1)		–	–	–	–	–	512 (83.8)	68 (11.1%)	396 (64.8)	184 (30.1%)	75 (12.3)	505 (82.6%)	–	–	–	–	–
	–	891 (74.9)	298 (25.1)		–	–	–	–	–	1028 (86.5)	100 (8.4%)	825 (69.4)	303 (25.5%)	96 (8.1)	1032 (86.8%)	–	–	–	–	–
Oberhauser 2020	12 (6.4)	138 (73.4)	27 (14.4)		2 (1.1)	8 (4.3)	–	–	–	–	–	–	–	9 (4.8)	–	9 (4.8)	–	91 (48.4)	40 (21.3)	15 (8.0)
	11 (11.6)	64 (67.4)	17 (17.9)		3 (3.2)	0	–	–	–	–	–	–	–	2 (2.1)	–	4 (4.2)	–	50 (52.6)	21 (22.1)	6 (6.3)
Rose 2019	–	101 (51.3)	74 (37.6)		9 (4.6)	9 (4.6)	–	–	–	165 (83.8)	29 (14.7)	79 (40.1)	28 (14.2)	22 (11.2)	169 (85.8)	–	–	–	–	–
	–	925 (66.1)	362 (25.9)		69 (4.9)	27 (1.9)	–	–	–	1205 (86.1)	188 (13.4)	490 (35.0)	231 (16.5)	120 (8.6)	1056 (75.5)	–	–	–	–	–
Rose 2020	–	–	–		–	–	–	–	–	–	–	–	–	–	–	–	–	–	–	–
	–	–	–		–	–	–	–	–	–	–	–	–	–	–	–	–	–	–	–
Sakina 2021	–	–	–		–	–	–	–	–	–	–	–	–	–	–	–	–	–	–	–
	–	–	–		–	–	–	–	–	–	–	–	–	–	–	–	–	–	–	–

Invasive ductal carcinoma (*IDC*), invasive lobular carcinoma (*ILC*), invasive ductal and lobular carcinoma (*IDLC*), ductal carcinoma in situ (*DCIS*), A = upper outer quadrant, B = upper inner quadrant, C = lower outer quadrant, D = lower inner quadrant, E = upper quadrants junction, F = lower quadrants junction, G = central quadrant

*Mixed type (DCIS + I)

### Risk of bias assessment:

Observational studies were assessed using the modified Newcastle–Ottawa scale (NOS) [14]. All of them showed high quality on this scale except Behluli et al. 2019 [18], that showed moderate quality (Table 3). The Cochrane Collaboration’s tool [12] revealed that Dogru et al. 2018 [22] had a lower risk of bias (Supplementary Fig. 1).

**Table 3:** Risk of bias for observational studies with the Newcastle–Ottawa Scale

Study ID	Study type	Criteria								Total	Quality
		1	2	3	4	5	6	7	8		
Atallah 2021	Case-control	1	1	0	1	1	1	1	1	7	High
Behluli 2019	Cohort	0	1	1	1	0	1	1	1	6	Moderate
Bromberg 2018	Cohort	0	1	1	1	1	1	1	1	7	High
Chauhan 2016	Cohort	0	1	1	1	1	1	1	1	7	High
Dogan 2021	Cohort	1	1	1	1	1	1	1	0	7	High
Gulcelik 2013	Cohort	1	1	1	1	1	1	1	1	8	High
Kelemen 2018	Cohort	1	1	1	1	1	1	1	1	8	High
Mátrai 2014	Cohort	1	1	1	1	1	1	1	1	8	High
Niinikoski 2019	Cohort	1	1	1	1	1	1	1	1	8	High
Oberhauser 2020	Cohort	1	1	1	1	1	1	1	1	8	High
Rose 2019	Cohort	1	1	1	1	1	1	1	1	8	High
Rose 2020	Cohort	1	1	1	1	1	1	1	0	7	High
Sakina 2021	Cohort	1	1	1	1	1	1	1	1	8	High

### Efficacy outcomes

#### Re-excision

**Fig. 2:**
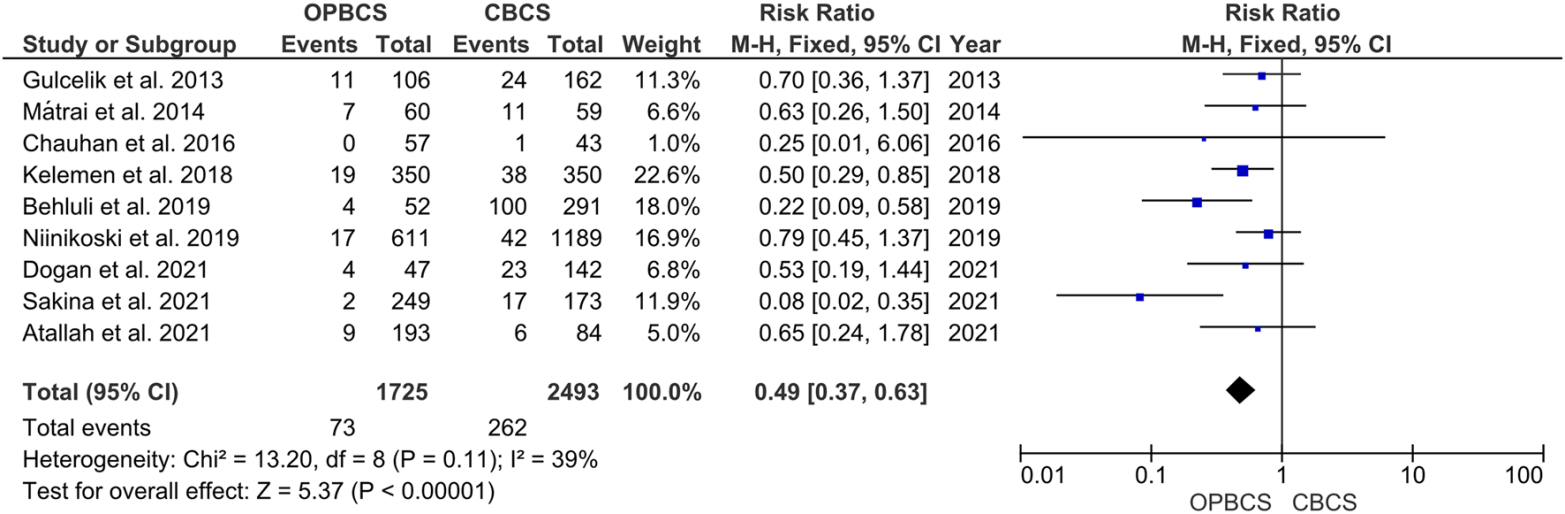
Forest plot of risk ratio (RR) for re-excision rate

The pooled analysis of the included studies showed a significant difference between both groups (RR = 0.49; 95% CI [0.37, 0.63]; *P* < 0.00001), favouring the OPBCS group over the CBCS group regarding the re-excision rates. The pooled studies in this outcome were homogenous, and little amounts of heterogeneity were detected between the included pooled studies (*P* = 0.11; *I*^2^ = 39%) (Fig. 2).

#### Local recurrence

**Fig. 3:**
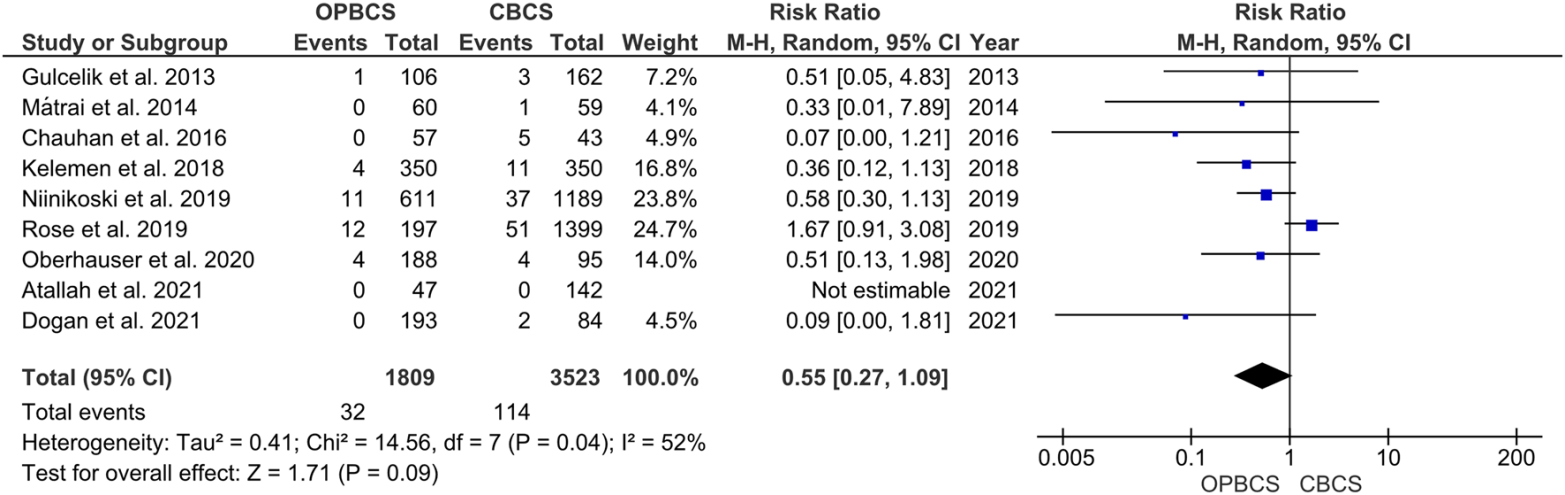
Forest plot of risk ratio (RR) for local recurrence

The pooled estimate of the included studies shows no significant difference between both groups in terms of local recurrence (RR = 0.55, 95% CI [0.27, 1.09], *P* = 0.09). Pooled studies were heterogeneous (*P* = 0.04; *I*^2^ = 52%), and the heterogeneity was best resolved by excluding Rose et al. [27] (*P* = 0.73; *I*^2^ = 0%), favouring OPBCS over CBCS (RR = 0.46, CI [0.28, 0.75], *P* = 0.002) (Fig. 3).

#### Positive surgical margin

**Fig. 4:**
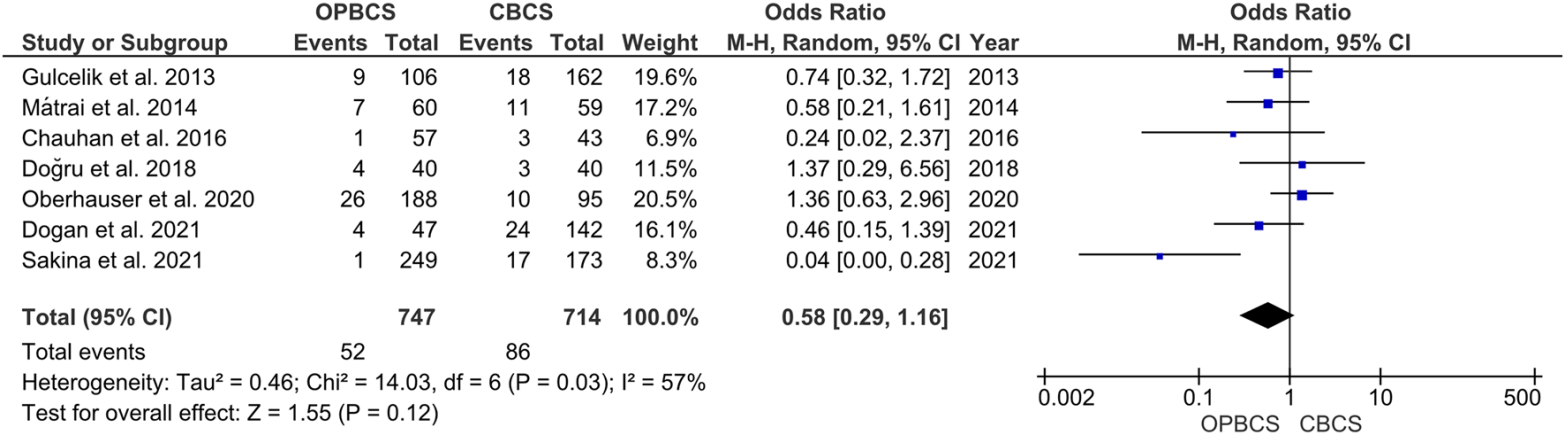
Forest plot of risk ratio (RR) for positive surgical margin

The pooled analysis showed no significant difference between both groups (RR = 0.58, 95% CI [0.29, 1.16], *P* = 0.12). The pooled studies were heterogeneous (*P* = 0.03; *I*^2^ = 57%), and the heterogeneity was best resolved by excluding Sakina et al. [29] (*P* = 0.43; *I*^2^ = 0%) favouring OPBCS over CBCS (RR = 0.70; CI [0.49, 1], *P* = 0.05) (Fig. 4).

#### Mastectomy

**Fig. 5:**
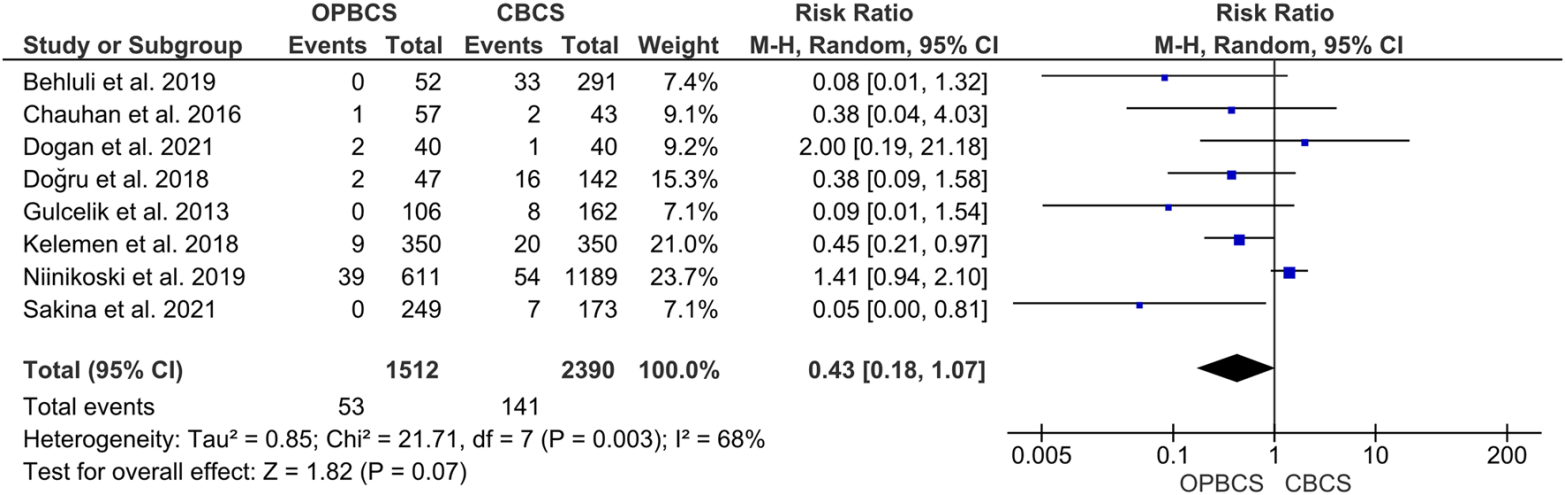
Forest plot of risk ratio (RR) for mastectomy

The pooled Risk ratio shows no significant difference between the OPBCS group and the CBCS group (RR = 0.43, 95% CI [0.18, 1.07], *P* = 0.07). Pooled studies reporting this parameter were heterogeneous (*P* = 0.003; *I*^2^ = 68%), and the heterogeneity was best resolved by excluding Niinikoski et al. [25] (*P* = 0.28; *I*^2^ = 19%) favouring OPBCS over CBCS (RR = 0.43, CI [0.23, 0.78], *P* = 0.005) (Fig. 5).

#### Reoperation

The pooled risk ratio shows no significant difference between the OPBCS group and the CBCS group (RR = 0.96; 95% CI [0.75, 1.25], *P* = 0.78), as shown in supplementary Fig. 2. Studies reporting this parameter were homogenous (*P* = 0.16; *I*^2^ = 46%).

#### Radiotherapy

As demonstrated in supplementary Fig. 3, the pooled risk ratio demonstrates that there was no statistically significant difference between the OPBCS and CBCS groups (RR = 1.02, 95% CI [1, 1.04], *P* = 0.05). Studies reporting this parameter were homogenous (*P* = 0.08; *I*^2^ = 49%).

#### Chemotherapy

As shown in supplementary Fig. 4, the pooled risk ratio favours the CBCS group over the OPBCS group (RR = 1.48, 95% CI [1.33, 1.64], *P* = 0.00001). Studies reporting this parameter were homogenous (*P* = 0.19; *I*^2^ = 32%).

#### Endocrine therapy

As demonstrated in supplementary Fig. 5, the pooled risk ratio demonstrates no significant difference between the OPBCS and CBCS groups (RR = 1.07, 95% CI [0.90, 1.27], *P* = 0.44). Studies reporting this parameter were heterogeneous (*P* < 0.0001; *I*^2^ = 85%). The heterogeneity could not be resolved by sensitivity analysis due to the high variability of the Mean RR in the included studies.

#### Immunotherapy

As illustrated in supplementary Fig. 6, the pooled risk ratio demonstrates no significant difference between the OPBCS and CBCS groups (RR = 1.21, 95% CI [0.82, 1.79], *P* = 0.34). Studies reporting this parameter were homogenous (*P* = 0.91; *I*^2^ = 0%).

#### Ipsilateral breast tumour recurrence

As demonstrated in supplementary Fig. 7, the pooled risk ratio demonstrates no significant difference between the OPBCS and CBCS groups (RR = 0.61, 95% CI [0.31, 1.21], *P* = 0.16). Studies reporting this parameter were homogenous (*P* = 0.69; *I*^2^ = 0%).

#### Surgical time (min)

As demonstrated in supplementary Fig. 8, the pooled risk ratio demonstrates no significant difference between the OPBCS and CBCS groups (RR = − 0.17, 95% CI [− 56.34, 55.99], *P* = 1.00). Studies reporting this parameter were heterogeneous (*P* < 0.00001; *I*^2^ = 91%). We resolved heterogeneity by removing Bromberg et al. 2018 [19] (*P* = 0.3; *I*^2^ = 7%), and there was also no significant difference between OPBCS and CBCS (RR = 17.51, CI [− 1.03, 36.04], *P* = 0.06).

#### Negative surgical margin

As indicated in supplementary Fig. 9, the pooled risk ratio demonstrates no significant difference between the OPBCS and CBCS groups (RR = 1.02, 95% CI [0.94, 1.11], *P* = 0.27). Studies reporting this parameter were heterogeneous (*P* = 0.08; *I*^2^ = 55%). The analysis was heterogeneous, but we resolved it by excluding Chauhan 2016 [20] (*P* = 0.2, *I*^2^ = 36%), and there was no significant difference between OPBCS and CBCS (RR = 1.02, CI [0.96, 1.08], *P* = 0.5).

#### Close surgical margins

As described in supplementary Fig. 10, the pooled risk ratio favours the OPBCS group over the CBCS group (RR = 0.37, 95% CI [0.14, 1.00], *P* = 0.05). Studies reporting this parameter were homogenous (*P* = 0.56; *I*^2^ = 0%).

#### Dissected lymph nodes

Supplementary Fig. 11 reveals that dissected lymph nodes were more common in the CBCS group than in the OPBCS group (RR = 1.22, 95% CI [1.07, 1.39], *P* = 0.002). Studies reporting this parameter were homogenous (*P* = 0.39; *I*^2^ = 0%).

### Safety outcomes

#### Total complication

**Fig. 6:**
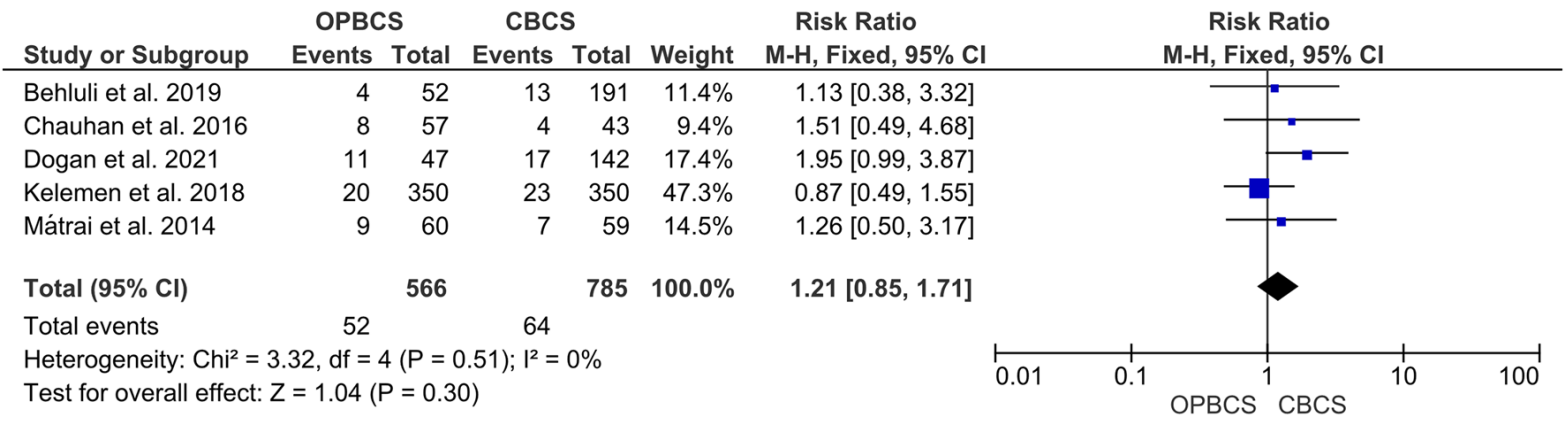
Forest plot of risk ratio (RR) for total complication

The pooled risk ratio demonstrates no significant difference between the OPBCS and CBCS groups (RR = 1.21, 95% CI [0.85, 1.71], *P* = 0.3). Studies reporting this parameter were homogenous (*P* = 0.51; *I*^2^ = 0%) (Fig. 6).

#### Hematoma

As indicated in supplementary Fig. 12, the pooled risk ratio demonstrates no significant difference between the OPBCS and CBCS groups (RR = 0.58, 95% CI [0.29, 1.16], *P* = 0.12). Studies reporting this parameter were homogenous (*P* = 0.99; *I*^2^ = 0%).

#### Nipple necrosis

As demonstrated in supplementary Fig. 13, the pooled risk ratio between the OPBCS and CBCS groups is not significantly different (RR = 1.87, % CI [0.20, 17.58], *P* = 0.58). Studies reporting this parameter were homogenous (*P* = 0.86; *I*^2^ = 0%).

#### Skin necrosis

As indicated in supplementary Fig. 14, the pooled risk ratio demonstrates no significant difference between the OPBCS and CBCS groups (RR = 1.45, 95% CI [4.00, 5.26], *P* = 0.58). Studies reporting this parameter were homogenous (*P* = 0.92; *I*^2^ = 0%).

#### Necrosis

As indicated in supplementary Fig. 15, the pooled risk ratio demonstrates no significant difference between the OPBCS and CBCS groups (RR = 2.6, 95% CI [0.72, 9.4], *P* = 0.15). Studies reporting this parameter were homogenous (*P* = 0.39; *I*^2^ = 0%).

#### Wound healing perturbation

Supplementary Fig. 16 shows that the pooled risk ratio favours the CBCS group over the OPBCS group (RR = 3.66, 95% CI [1.43, 9.33], *P* = 0.007). Studies reporting this parameter were homogenous (*P* = 0.82; *I*^2^ = 0%).

#### Seroma

As indicated in supplementary Fig. 17, the pooled risk ratio demonstrates no significant difference between the OPBCS and CBCS groups (RR = 1.17, 95% CI [0.64, 2.13], *P* = 0.62). Studies reporting this parameter were homogenous (*P* = 0.35; *I*^2^ = 10%).

#### Infection

As shown in supplementary Fig. 17, the pooled estimate revealed the risk ratio between the OPBCS and CBCS groups was not significantly different (RR = 1.19, 95% CI [0.63, 2.26], *P* = 0.58). Studies reporting this parameter were homogenous (*P* = 0.97; *I*^2^ = 0%).

#### Death

The pooled analysis revealed that the risk ratio between the OPBCS and CBCS groups was not significantly different (RR = 0.96, 95% CI [0.53, 1.73], *P* = 0.89), as shown in supplementary Fig. 19. Studies reporting this parameter were homogenous (*P* = 0.25; *I*^2^ = 27%).

## Discussion

Breast cancer and plastic surgeons have adapted and used well-established aesthetic mammoplasty procedures to improve CBCS during the last ten years. This approach is at one end of the spectrum of what is now known as oncoplastic breast-conservation surgery (OPBCS) [10, 30].

Our MA analyzed 14 outcomes in the included studies, although all studies did not necessarily report all 14 outcomes. Among these outcomes, the most reported two were the re-excision and local recurrence rates, as they are cornerstone points in oncological breast surgery. Regarding re-excision, our MA showed that there is a significant difference in the re-excision between CBCS and OPBCS as the re-excision rates were lower in patients with OPBCS; and this makes sense as quadrantectomy is oncologically better than extensive local excision (‘lumpectomy’), as evidenced by large RCTs, but the cost is a major aesthetic deformity 34, 35. So, by permitting larger excision volumes and broader margins, OPBCS utilizing reduction mammoplasty procedures may be oncologically superior to CBCS. The low re-excision rates in OPBCS in our MA enhance this superiority [31–34]. Kelemen et al., Behluli et al., and Sakina et al. reported lower re-excision rates in the OPBCS group [18, 23, 29]. However, Gulcelik et al., Matrai et al., Chauhan et al., Niinikoski et al., Dogan et al., and Atallah et al. show no significant difference in the re-excision rates [14, 17, 20, 21, 24, 25].

We also found that the local recurrence rate was lower in the OPBCS group after resolving heterogeneity by removing Rose et al. 2019 [27] as there was a big difference in the number of patients in the CBCS group (1399) versus the OPBCS group (197) before resolving heterogeneity there was no difference between OPBCS and CBCS groups. Gulcelik et al., Matrai et al., Chauhan et al., Atallah et al., Niinikoski et al., Kelemen et al. and Oberhauser et al. have lower rates of recurrence in OPBCS groups compared with CBCS groups, but the difference between two groups in each study individually isn't statistically significant [14, 17, 20, 23–26]. Losken et al. also reported a low recurrence rate in OPBCS compared to CBCS. [33] A low local recurrence rate makes sense, as OPBCS allows wide excisions with good aesthetic outcomes. [33] In addition to local recurrence, we also assessed ipsilateral breast cancer recurrence, and there is no significant difference between OPBCS and CBCS. Niinikoski et al. [25] and Dogan et al. [21] show no difference between CBCS and OPBCS regarding ipsilateral breast recurrence.

In our MA, dissected lymph nodes were more in CBCS than OPBCS. Rose et al. 2019 [27] and Rose et al. 2020 [28] also showed that dissected lymph nodes were more in the CBCS group, but Gulcelik et al. [14] and Niinikoski et al. [25] stated that there is no difference between the two groups.

Mastectomy in our study was lower in the OPBCS group after resolving heterogeneity by removing Niinikoski et al. [25], as there was a big difference in the number of patients in the CBCS group (1189) versus the OPBCS group (611) before resolving heterogeneity, there was no difference between OPBCS and CBCS groups. Behluli et al., Chauhan et al., Dogan et al., Doğru et al., Gulcelik et al., Kelemen et al., and Sakina et al. have lower rates of mastectomy in OPBCS groups compared with CBCS groups. Still, the difference between the two groups in each study individually is not statistically significant [14, 18, 20–23, 29]. On the contrary, Niinikoski et al. [25] have higher mastectomy rates in the CBCS group than the OPBCS group, but the difference between the two groups is not statistically significant. However, Losken et al. [33] found that even though the incidence of positive margins is lower in the oncoplastic groups, patients who have positive margins are more likely to have a complete mastectomy rather than re-excision, so when a wide resection is performed, and positive margins persist, the patient may no longer be a candidate for breast preservation, and a complete mastectomy becomes the logical next step.

We also assessed surgical time in our analysis, and there is no significant difference between the OPBCS group and the CBCS group. The analysis was heterogeneous, and the heterogeneity was best resolved by excluding Bromberg et al. [19], and after heterogeneity was resolved, the difference between OPBCS and CBCS was still not significant. Behluli et al. [18] also has no significant difference between OPBCS and CBCS in the surgical time but in Bromberg et al. [19]. Surgical time was lower in the OPBCS group and in Matrai et al. [24] it was lower in the CBCS group.

In our MA, we assessed positive and negative surgical margins, and there is not a significant difference between OPBCS and CBCS. The studies were heterogeneous, but the heterogeneity was resolved in the positive surgical margin by excluding Sakina et al. [29] and in the case of negative surgical margin by excluding Chauhan et al. [20] still, even after removing heterogeneity, there is no significant difference between the two groups. In Gulcelik et al. [14], Mátrai et al. [24], Chauhan et al. [20], Doğru et al. [22], Oberhauser et al. [26], Dogan et al. [21] there is no significant difference between positive surgical margins neither in OPBCS or CBCS. On the contrary, in Sakina et al. [29] there are fewer positive surgical margins in OPBCS groups. In Losken et al. [33] the positive surgical margins were fewer in the OPBCS group, which could be explained by wider excision in OPBCS. Doğru et al. [22], Oberhauser et al. [26], and Atallah et al. [17] found that there is no difference between negative surgical margins between OPBCS and CBCS, but in Chauhan et al. [20] there is more negative margins in OPBCS than CBCS, and this also explained by wider excision in OPBCS.

Our analysis also concluded that patients who have OPBCS have less close surgical margins than CBCS, which is also explained by the wider excision that occurs in OPBCS. Gulcelik et al. [14], Doğru et al. [22] and Chauhan et al. [20] found that there is no difference regarding close surgical margins,

Regarding adjunctive Radiotherapy after OPBCS or CBCS, there is no significant difference between the two groups; also, Atallah et al. [17], Mátrai et al. [24], Niinikoski et al. [25], Oberhauser et al. [26], Rose et al. 2019 [27] and Rose et al. 2020 [28] found that there is no difference in adjunctive radiotherapy therapy after two operations. Conversely, adjunctive Chemotherapy is needed more in the CBCS group compared to OPBCS. Mátrai et al. [24] Niinikoski et al. [25], Oberhauser et al. [26], Rose et al. 2019 [27] and Gulcelik et al. [14] found that Chemotherapy was needed more in the CBCS group, but Oberhauser et al. [26] and Rose et al. 2020 [27] show no significant difference between two groups.

In this analysis, there are three studies reporting Reoperation, and we found no significant difference between OPBCS and CBCS. Niinikoski et al. [25], Chauhan et al. [20], and Kelemen et al. [23] show no significant difference between OPBCS and CBCS.

Regarding endocrine therapy, there is no significant difference between OPBCS and CBCS; the studies included were heterogeneous, but the heterogeneity could not be resolved by sensitivity analysis due to the high variability of the Mean RR in the included trials. Mátrai et al. [24], Niinikoski et al. [25], Rose et al. 2019 [27] and Rose et al. 2020 [28] also don’t find any significant difference between OPBCS and CBCS regarding endocrine therapy, but in Gulcelik et al. [14] adjunctive endocrine therapy used more in the CBCS group.

Our MA also assessed immune therapy and showed no significant difference between OPBCS and CBCS. Rose et al. 2019 [27] and Rose et al. 2020 [28] show no significant difference between the two groups.

In our study, we also assessed complications reported in our included studies. We found no significant difference between OPBCS and CBCS in the following complications: Hematoma, Necrosis, skin necrosis, nipple necrosis seroma, Infection, Death and total complication. Only wound healing perturbation shows a significant difference towards the CBCS group.

Our study contains many patients (exactly 6941 patients) that were included in all studies. We assessed 14 outcomes and nine complications versus six outcomes and complications on Losken et al. [33] Also, Losken et al. [33] collected data from separate series in each group CBCS or OPBCS. Still, in our MA, the data were collected from comparative studies (RCTs), cohort and case-control studies. Data are homogenous except for five heterogeneous outcomes, and this is resolved by applying a random effect model and then excluding one study. Our data was extracted from different studies conducted in various centres and surgeons, so we may need more studies that stratified these factors as OPBCS requires combined skills, knowledge, and understanding of oncological and plastic surgeries, so variability in the previous factors may affect results. Also, we need more studies that stratify and analyze different approaches and techniques of OPBCS as volume displacement and volume replacement.

In conclusion, oncoplastic surgery attempts to provide both oncological and aesthetic benefits. OPBCS appears to be more effective than CBCS in terms of re-excision rate and local recurrence, close surgical margins, and mastectomy. Using an oncoplastic technique, a bigger volume resection allows for a complete assessment of the surrounding breast tissue. In terms of complication, there was no significant difference between the OPBCS and CBCS groups, except for wound healing perturbation, which was higher in the CBCS groups.

## Supplementary Information

Below is the link to the electronic supplementary material.

Supplementary file1 (DOCX 192 KB)
