# Supplementary material for: Oncoplastic versus conventional breast-conserving surgery in breast cancer: a pooled analysis of 6941 female patients
Source: Breast Cancer. 2023 Jan 9;30(2):200–14. doi: 10.1007/s12282-022-01430-5 (PMC9950210; doi:10.1007/s12282-022-01430-5)

**Supplementary 1**

**Supplementary Figure 1:** Cochrane risk of bias summary risk of bias graph and figure for Dogru et al. 2018


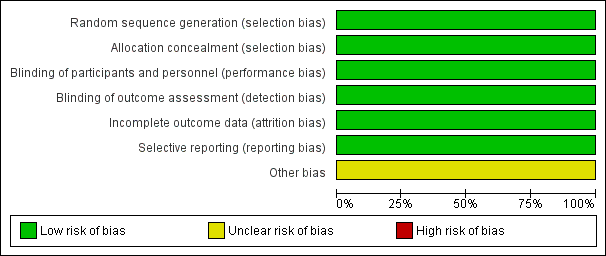

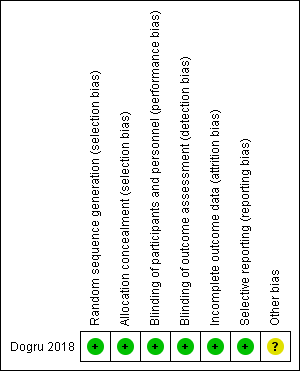


***Supplementary F*igure 2.** Forest plot of risk ratio (RR)for Reoperation rate


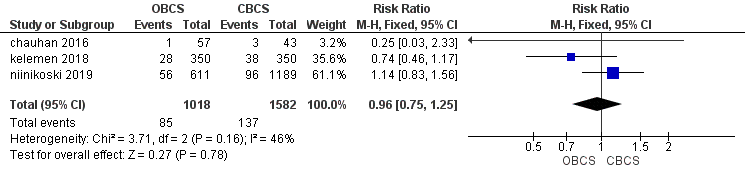


***Supplementary F*igure 3.** Forest plot of risk ratio (RR)for Radiotherapy


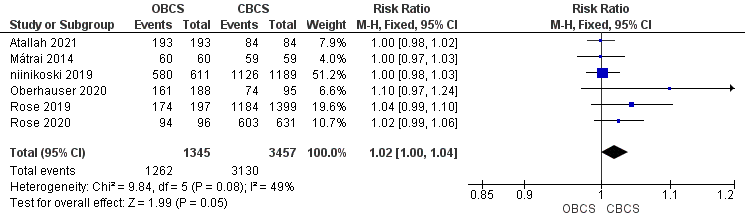


***Supplementary F*igure 4.** Forest plot of risk ratio (RR)for Chemotherapy


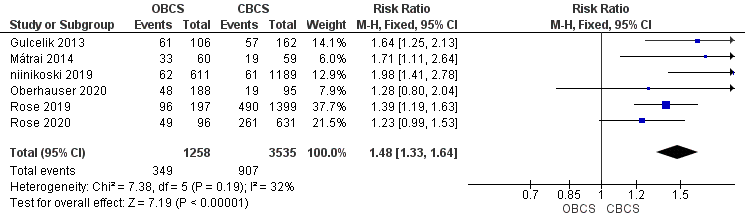


**Supplementary Figure 5.** Forest plot of risk ratio (RR)for Endocrine therapy


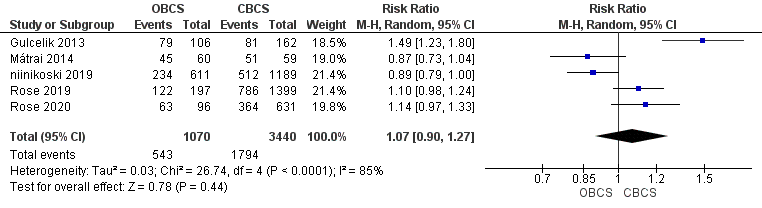


**Supplementary Figure 6.** Forest plot of risk ratio (RR)for Immune therapy


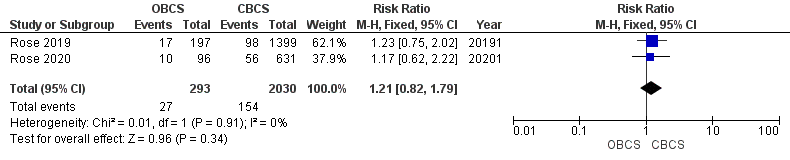


***Supplementary F*igure 7.** Forest plot of risk ratio (RR)for Ipsilateral tumor recurrence


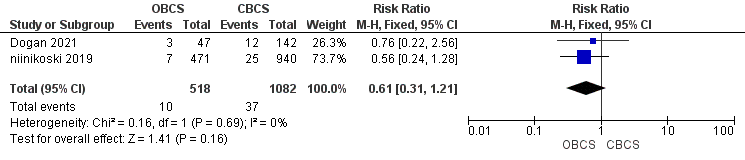


***Supplementary F*igure 8.** Forest plot of risk ratio (RR)for Surgical time


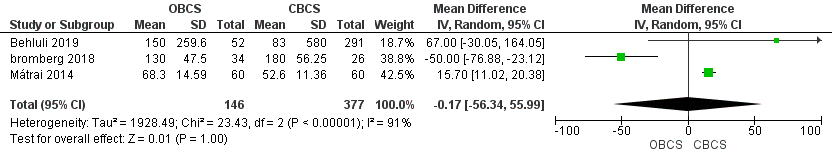


**Supplementary Figure 9.** Forest plot of risk ratio (RR)for Negative Surgical margin


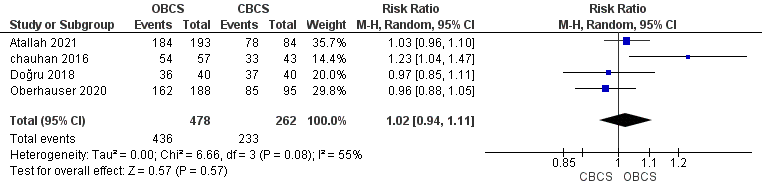


**Supplementary Figure 10.** Forest plot of risk ratio (RR)for Close surgical margin


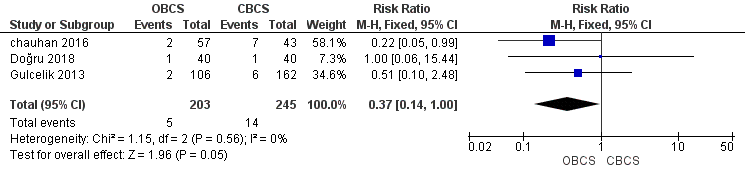


**Supplementary Figure 11.** Forest plot of risk ratio (RR)for Dissected lymph nodes


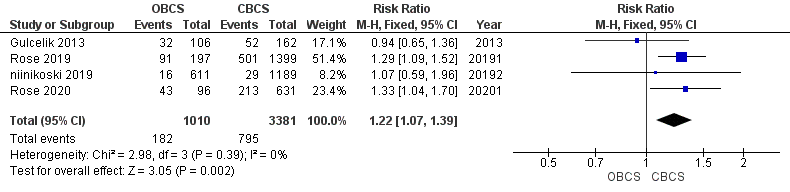


***Supplementary F*igure 12.** Forest plot of risk ratio (RR)for Hematoma


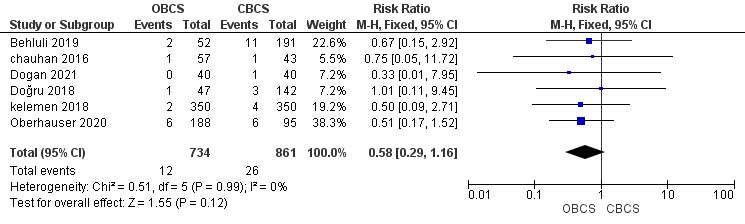


***Supplementary F*igure 13.** Forest plot of risk ratio (RR)for Nipple necrosis


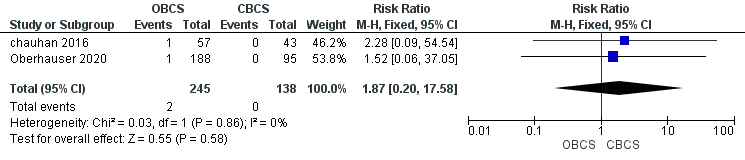


**Supplementary Figure 14:** Forest plot of risk ratio (RR)for Skin necrosis


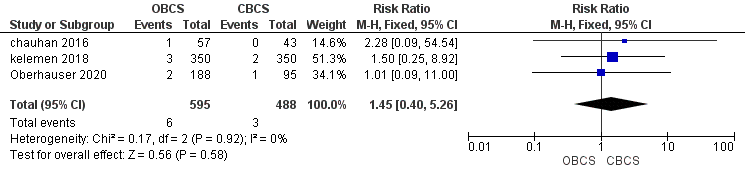


**Supplementary Figure 15:** Forest plot of risk ratio (RR)for Overall Necrosis


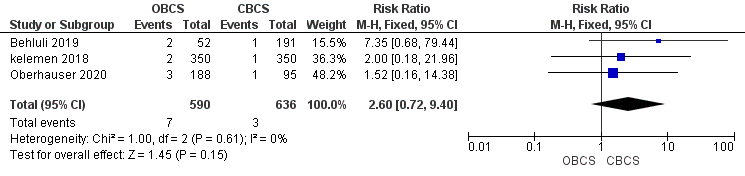


**Supplementary Figure 16:** Forest plot of risk ratio (RR)for Wound healing perturbation


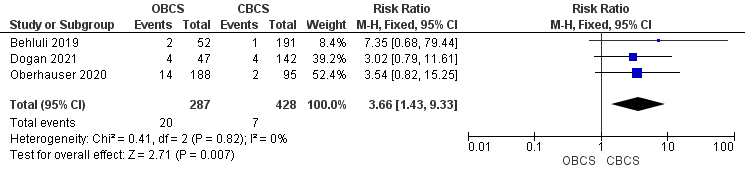


**Supplementary Figure 17:** Forest plot of risk ratio (RR)for Seroma


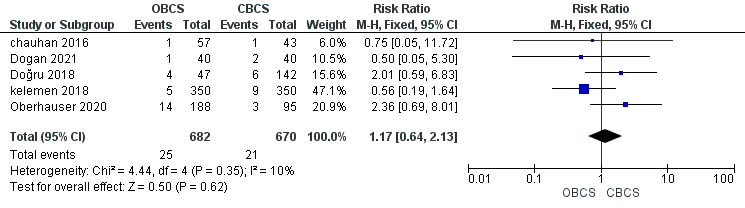


**Supplementary Figure 18:** Forest plot of risk ratio (RR)for Infection


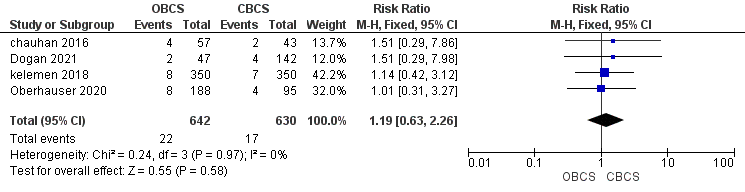


***Supplementary* Figure** 19: Forest plot of risk ratio (RR)for Death


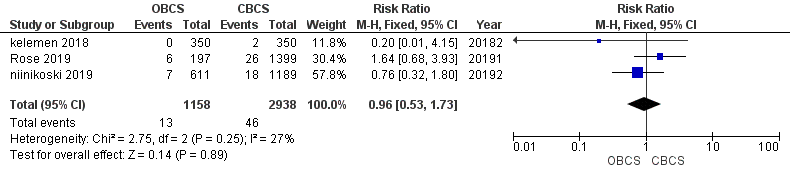

Supplement: Supplementary file 1 — Supplementary file1 (DOCX 192 KB) [file 12282_2022_1430_MOESM1_ESM.docx]
